# The Cream of the Crop of the Medicinal Chemistry Section of *Molecules*—2019

**DOI:** 10.3390/molecules26041053

**Published:** 2021-02-17

**Authors:** Diego Muñoz-Torrero

**Affiliations:** Laboratory of Medicinal Chemistry (CSIC Associated Unit), Faculty of Pharmacy and Food Sciences, and Institute of Biomedicine (IBUB), University of Barcelona, Av. Joan XXIII, 27-31, E-08028 Barcelona, Spain; dmunoztorrero@ub.edu

The MDPI journal *Molecules* is organized into 25 sections that cover many different areas of the broad field of chemistry. Among them, the Medicinal Chemistry section is one of the most classical. The overarching aim of this section is the publication of original re-search and review articles that increase our knowledge on how the chemical structure of molecules can be advantageously modulated to enhance their physicochemical and pharmacokinetic properties and/or their interaction with particular biological targets of therapeutic or diagnostic interest. With Medicinal Chemistry being multidisciplinary in essence, the contents of the articles published in this section partly overlap with those of other sections—namely, organic, natural products, bioorganic, and computational theo-retical chemistry or chemical biology, which contributes to the integration and coherence among the different areas of the journal.

Driven by a very active board of Academic Editors and imaginative section Managing Editors, new actions are continuously undertaken to attract, visualize, and promote high quality articles and reviews, with the final goal of disseminating in open access medicinal chemistry of excellence. On this line, we are initiating a series of Editorials to highlight the most influential articles, overall or considering specific topics, published in the Medicinal Chemistry section of *Molecules* in a particular year. We will start with the articles published in 2019, now that all of them have had an exposure to readers of at least one year.

To contextualize this action in the situation of *Molecules* in that year, in 2019:

- *Molecules* started to be published semi-monthly online.
- The scope of the journal was expanded by establishing the new sections of Fragances and Flavours and Colorants.
- The important milestone of reaching 20,000 articles published in the journal was achieved.
- The 2nd *Molecules* Medicinal Chemistry Symposium (MMCS2019—Facing Novel Challenges in Drug Discovery), organized by the Medicinal Chemistry Section of *Molecules*, was successfully held in Barcelona, with a 3-day program full of top-class speakers and about 200 attendees from all continents.
- For 2019, *Molecules* reached its highest ever impact factor of 3.267.
- With regard to the status of the Medicinal Chemistry section of *Molecules*, in 2019:
- A record of submissions (1197) and published articles (583) was achieved, consolidating a steady increase over previous years (Figure 1). Out of the 583 published papers, 482 were original research articles, but a significant number of review articles were also published (81), as well as a lower number of communications (10), editorials (7), and other types of contributions (commentaries, corrections) to complete the aforementioned figure.

- The top 10 contributing countries, considering the affiliation of the corresponding authors of all articles published in the section in 2019, were China, USA, Italy, Poland, France, Germany, Korea, Spain, Japan, and Saudi Arabia (Figure 2).

- The section experienced a large increase in the number of special issues launched in 2019, relative to the previous four years, almost doubling those launched in 2018 (Figure 3).

A total of 644 articles have been published in the 113 Special Issues launched in 2019, with some of them having been published in 2020 in the case of Special Issues with submission deadlines beyond 2019. Overall, an average of 5.7 articles have been published per Special Issue launched in 2019. Out of the 113 Special Issues launched in 2019, 24 gathered 10 or more articles. Without considering the period of time for submission of manuscripts, which can vary significantly among Special Issues, the top 10 Special Issues of 2019 in which more articles have been published were:

**Anticancer Agents: Design, Synthesis and Evaluation**, Qiao-Hong Chen (Ed.), 32 papers.

**Recent Advances in Anticancer Drugs II**, Simona Collina and Mariarosaria Miloso (Eds.), 27 papers.

**Advances in Nucleoside/Nucleotides and Nucleic Acid Chemistry: A Theme Issue in Honor of Prof. Dr. Piet Herdewijn**, Katherine Seley-Radtke (Ed.), 24 papers.

**Recent Trends on Enzymes Inhibitors and Activators in Drug Research II**, Athina Geronikaki (Ed.), 22 papers.

**Anticancer Drug Discovery and Development**, Bartosz Tylkowski, Anna Bajek, and Krzysztof Roszkowski (Eds.), 20 papers.

**Opioids and Their Receptors: Present and Emerging Concepts in Opioid Drug Discovery**, Mariana Spetea and Helmut Schmidhammer (Eds.), 19 papers.

**Biological Activity of Natural Substances and Their Derivatives**, Olga Pecháňová and Martina Cebova (Eds.), 18 papers.

**Bioconjugation Strategies in Drug Delivery and Molecular Imaging**, Andrea Trabocchi and Elena Lenci (Eds.), 16 papers.

**Advances in Anticancer Drug Discovery**, Jóhannes Reynisson (Ed.), 15 papers.

**Translational Approach to Antitumor Drugs**, Krešimir Pavelić and Sandra Kraljevic Pavelic (Eds.), 14 papers.

- As a final remark, I would like to highlight the impressive number of views (752,853) and downloads (680,386) that the 583 articles published in the section in 2019 have received so far, which leads to an average of 1291 views and 1167 downloads per article. The evident interest that all these articles have generated in the scientific community is backed the high number of citations (2939) they have received thus far.

Not without first recognizing the high quality of all the articles published in the Medicinal Chemistry section of *Molecules* in 2019 and thanking all the authors for their trust in the section, a limited number of original research articles and review articles have been selected on the basis of the number of citations and downloads and are listed below in Table 1 and Table 2.

## Figures and Tables

**Figure 1:**
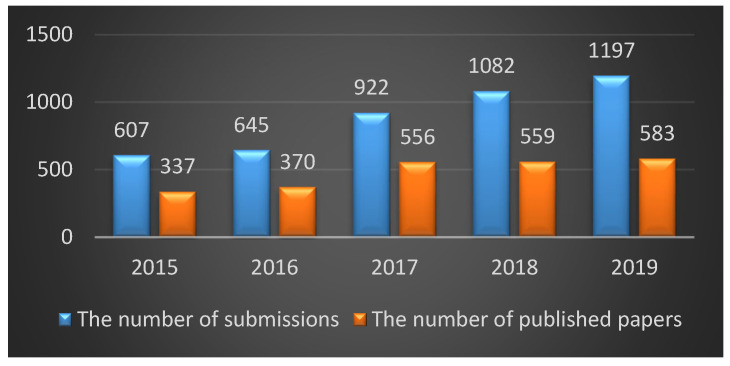
Number of submitted manuscripts (blue bars) and published papers (orange bars) in the Medicinal Chemistry section of *Molecules* in the period 2015–2019.

**Figure 2:**
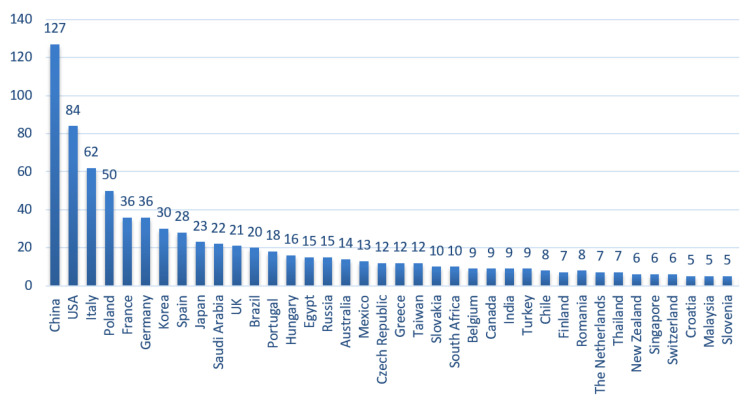
Geographical distribution of the corresponding authors of the papers published in the Medicinal Chemistry section of *Molecules* in 2019. The number of published articles is indicated on top of the bars. Only countries with five or more contributions are shown.

**Figure 3:**
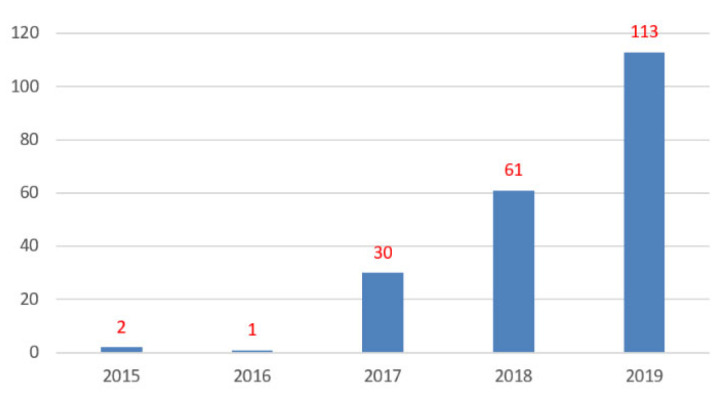
Total number of special issues launched in the Medicinal Chemistry section of *Molecules* in 2019.

**Table 1:** The top 10 articles of 2019 in the Medicinal Chemistry section of *Molecules.*

Title	Authors	References
CA-170—A Potent Small-Molecule PD-L1 Inhibitor or Not?	Bogdan Musielak et al.	[1]
ACPred: A Computational Tool for the Prediction and Analysis of Anticancer Peptides	Nalini Schaduangrat et al.	[2]
1,4-Naphthoquinone Analogues: Potent Antibacterial Agents and Mode of Action Evaluation	Palanisamy Ravichandiran et al.	[3]
Preparation, Characterization, and Release Kinetics of Chitosan-Coated Nanoliposomes Encapsulating Curcumin in Simulated Environments	Mahmood Hasan et al.	[4]
Identification of High-Affinity Inhibitors of Cyclin-Dependent Kinase 2 Towards Anticancer Therapy	Taj Mohammad et al.	[5]
Synthesis and Biological Evaluation of Quinoline Derivatives as a Novel Class of Broad-Spectrum Antibacterial Agents	Hai-Gen Fu et al.	[6]
Modeling Physico-Chemical ADMET Endpoints with Multitask Graph Convolutional Networks	Floriane Montanari et al.	[7]
A Machine Learning Approach for the Discovery of Ligand-Specific Functional Mechanisms of GPCRs	Ambrose Plante et al.	[8]
Incorporation of Putative Helix-Breaking Amino Acids in the Design of Novel Stapled Peptides: Exploring Biophysical and Cellular Permeability Properties	Anthony W. Partridge et al.	[9]
A Repurposing Approach for Uncovering the Anti-Tubercular Activity of FDA-Approved Drugs with Potential Multi-Targeting Profiles	Basem Battah et al.	[10]

**Table 2:** The top 10 review articles of 2019 in the Medicinal Chemistry section of *Molecules.*

Title	Authors	References
Oxidative Stress: A Key Modulator in Neurodegenerative Diseases	Anju Singh et al.	[11]
Recent Progress of Targeted G-Quadruplex-Preferred Ligands Toward Cancer Therapy	Sefan Asamitsu et al.	[12]
The Pharmaceutical Industry in 2018. An Analysis of FDA Drug Approvals from the Perspective of Molecules	Beatriz G. de la Torre and Fernando Albericio	[13]
Chitosan-Based (Nano)Materials for Novel Biomedical Applications	Gregor Kravanja et al.	[14]
Cannabinoid Signaling in the Skin: Therapeutic Potential of the “C(ut)annabinoid” System	Kinga Fanni Tóth et al.	[15]
Bee Venom: Overview of Main Compounds and Bioactivities for Therapeutic Interests	Rim Wehbe et al.	[16]
Peptide Conjugates with Small Molecules Designed to Enhance Efficacy and Safety	Rongjun He et al.	[17]
DHFR Inhibitors: Reading the Past for Discovering Novel Anticancer Agents	Maria Valeria Raimondi et al.	[18]
Autophagy Modulation as a Treatment of Amyloid Diseases	Zoe Mputhia et al.	[19]
Siderophore–Antibiotic Conjugate Design: New Drugs for Bad Bugs?	Kokob H. Negash et al.	[20]
